# Evaluation of Effectiveness of Vitamins C and E on Prevention of Renal Scar due to Pyelonephritis in Rat

**DOI:** 10.1155/2011/489496

**Published:** 2010-12-12

**Authors:** Fatemeh Emamghorashi, Seyed Mohamad Owji, Mohammad Motamedifar

**Affiliations:** ^1^Department of Pediatrics, Shiraz Nephrology Urology Research Center, Jahrom University of Medical Sciences Jahrom, Iran; ^2^Department of Pathology, Shiraz Medical School, Shiraz University of Medical Sciences Shiraz, Iran; ^3^Department of Bacteriology and Virology, Medical School, Shiraz HIV/Aids Research Center (SHARC), Shiraz University of Medical Sciences, Shiraz, Iran

## Abstract

*Aim* was evaluation of the effects of cosupplementation of, vitamins E and C, in preventing renal scarring in acute pyelonephritis. *Animals and Treatments*. Sixty rats were used, bacteria was injected through kidney. The rats were arranged randomly in 3 groups of 20 rats each. Rats in groups 1 and 2 were given once-daily intraperitoneal injections of gentamicin for ten consecutive days, beginning on the third day after inoculation. In group 2, vitamins E and C cotreatment and in group 3, vitamins E and C cotreatment without gentamicin injection were started. Three rats in each group were killed 24 hours after the inoculation (for infection and inflammation document) and forty-eight hours after the antibiotic injection (for efficacy of treatment). After eight weeks, the rest of rats were killed, and kidneys evaluated for percent of scaring. *Result*. There was also significant difference of degree of scar formation (1.4 and 3.4% versus 8.6%, *P* = .001). The group which received gentamicin only had moderate to severe scaring, but the two groups which received vitamin C and vitamin E showed no or mild renal scaring. *Conclusion*. The study showed that administration of antioxidants can protect scaring due to pyelonephritis with or without antibiotic administration.

## 1. Introduction

*Escherichia coli* strains that cause acute pyelonephritis possess special combinations of virulence factors. It has been suggested that bacterial characteristics and the host defense play significant roles in the development of the scars [1, 2]. 

It has also been proposed that oxygen-free radicals play a role in renal scar formation after an acute PNP model in monkeys and mice [3, 4]. Recent experimental studies demonstrate that oxygen-free radical scavengers and antioxidants can reduce tissue damage and renal scaring during acute and chronic PNP [4–7].

Antioxidant vitamins (A, E, C) increase tissue protection from oxidative stress [8]. Recently, it has been shown that both vitamins E and C decrease lipid peroxidation and augment the activity of antioxidant enzymes in the kidneys of diabetic rats [9]. Single-dose administration of vitamin E has had protective effects on cisplatin-induced nephrotoxicity in developing rats [10]. Administration of vitamin E following the onset of fever can reduce renal damage in pyelonephritis [11]. There have been several studies in recent years suggesting more effectiveness of combination therapy by cosupplementation of two antioxidants [12–14]. The aim of the present study was to evaluate the effects of cosupplementation of vitamins E and C in prevention of renal scarring in acute pyelonephritis.

## 2. Materials and Methods

### 2.1. Animals and Treatment

Sixty female Sprague-Dawley rats weighing 170–300 g (209.6 ± 25.3 grams) and with average of 12 weeks old were obtained from Laboratory Animal House of Shiraz University of Medical Sciences, Shiraz, Iran. They had free access to food and water throughout the experiment. Rats were arranged randomly in 3 groups of 20 rats as follows.

**Group 1**

Rats were given once-daily intraperitoneal injections of gentamicin (10 mg/kg) for five consecutive days, beginning on the third day after inoculation.

**Group 2**

Rats were given once-daily intraperitoneal injections of gentamicin for five consecutive days, beginning on the third day after inoculation, one dose of vitamin E (100 g body weight^−1^) was injected intramuscular, and vitamin C (200 mg l^−1^) was added totheir drinking water for 3 days.

**Group 3**

One dose of vitamin E (100 g body weight^−1^) was injected intramuscular, and vitamin C (200 mg  l^−1^) was added totheir drinking water for 3 days.

**Group 4**

These were normal rats without procedure for control of renal pathology, BUN and creatinine level as control group.

Totally, the design of this experiment was approved by Ethic Committee of Shiraz University of Medical Sciences.

### 2.2. Pyelonephritis Model

The bacterial strain used to induce pyelonephritis was *Escherichia coli* (H2O6, pap+), gentamicin sensitive that was obtained from patient with pyelonephritis. Before the induction of pyelonephritis, animals were anesthetized. The side of the animals was shaved and asepticzed, and a small incision was made at the level of the kidney. The left kidney was exposed, and 0.05 mL of an inoculum containing 10^9^ bacteria was injected through the upper left kidney. This technique, described previously by Kaye [15], produces a constant and severe pyelonephritis in the left kidney with extensive inflammation and abscess formation induced by the direct inoculation of *E. coli*. 

Forty-eight hours after the inoculation (for infection and inflammation document) and forty-eight hours after the antibiotic injection (for efficacy of testament), a minimum of 6 rats per group, and the rest of the rats after 8 weeks, were anesthetized and were killed by decapitation. A midline abdominal incision was made, and both kidneys were aseptically removed, decapsulated, and weighed. Left kidneys were halved, weighted, and sent for microbiology and pathology study. For microbiology study, the samples were homogenized in 3 mL of sterile saline at 4°C. Appropriate dilutions of homogenized kidneys were made, and 10-*μ*l samples were placed in triplicate on MacConkey agar. The numbers of CFU of *E. coli* in the kidneys were determined after an incubation of 18 h at 37°C (the CFU per milliliter of homogenate were transformed into CFU per gram of tissue). The bacterial enumeration was done at the dilution that allowed us to detect between 30 and 300 CFU/g of kidney. The limit of detection was 30 CFU/g of kidney. Kidneys were considered sterile when no CFU were detected on the agar.

### 2.3. Histology

Kidney tissues were harvested from scarified animals and fixed in 10% neutral formalin solution, stain with hematoxylin eosin (HE) and Masson. The preparations were evaluated with bright field microscope and were photographed. Microscopic renal lesions were scored on plastic sections at a magnification of ×400. Each slide was coded so that identification of the groups was not possible for the observer. Slices came from three different pieces of renal cortex for each rat. Cortex and medulla including glomerulus, tubules, vessels, and interstitium were evaluated. The percent of cellular infiltration in each section was considered as severity of inflammation (IIC: interstitial inflammatory cells). Severity of scar formation was classified based on percent of scaring as follows: zero percent for no scar, less than 5% mild, 5%–15% moderate, and more than 15% severe. 

At the end of experiment, rats in all groups were sacrificed under anesthesia, and blood samples were collected by cardiac puncture. Blood urea nitrogen (BUN) and serum creatinine were also measured in the end of study.

### 2.4. Statistics

 All statistical analyses were performed with the SPSS version 11.5. The data were expressed as mean ± SD and analyzed by ANOVA to determine the statistical significance of the difference between groups. A *P* value less than  .05 (*P* < .05) was considered significant.

## 3. Results

Sixty female Sprague-Dawley rats weighing 170–300 g (209.6 ± 25.3 grams) were studied. There was no significant difference between mean weights of rats in the groups (Table 1). The table also showed comparison between the groups according to BUN, creatinine, and left kidney weight. Mean creatinine level was lower in groups that received vitamin E and vitamin C (with or without antibiotic) (*P* < .005). Mean weight of left kidneys were lower in the groups which received gentamicin without vitamins.

### 3.1. Microbiological Finding

 Two days after organism inoculation, the cultures of renal tissue were positive. Table 1 showed colony count per milliliter of homogenate and per gram kidney. There was no significant different between the three groups according to severity of infection.

### 3.2. Histological Finding

**Figure 1:**
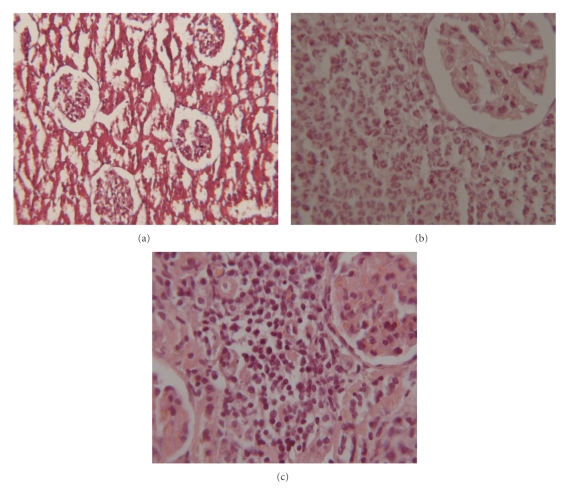
Kidney histology of rats in evaluation of inflammation: (a) normal, (b) severe acute inflammation 48 hours after inoculation, (c) and chronic inflammation (H&E, magnification of 400).

 Renal tissues were evaluated for severity of inflammation and scar formation. Renal pathology 48 hours after organism inoculation showed moderate to severe inflammation in the three groups; there was severe interstitial mononuclear cell infiltration. Chronic inflammation was observed in all rats in group 1 after eight weeks but not observed in groups 2 and 3. In controlled group, histology of kidney was normal (Figure 1). 

**Figure 2:**
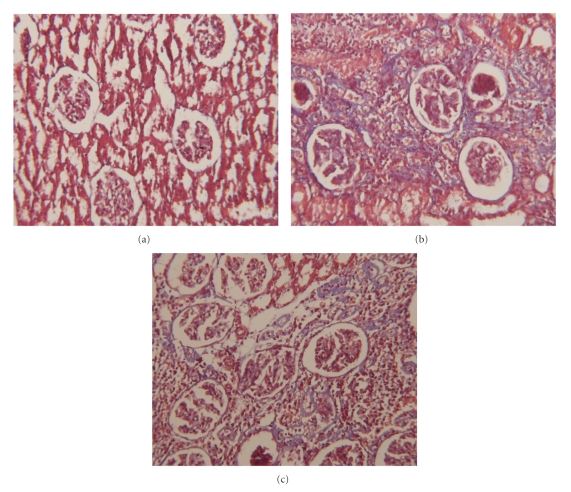
Kidney histology of rats for evaluation of severity of renal scaring: (a) normal, (b) the group without vitamin supplement showed severe scaring, and (c) the groups that received vitamin E and C showed mild scaring (Masson, magnification of 160).

There was significant difference in percentage of scar formation in groups that received vitamins E and C in comparison to group without them (*P* = .001). In groups that received vitamins E and C, severity of degenerative changes were less than the group without vitamin supplement. The group that did not receive vitamins E and C showed mean 8.6% scar formation (range 0%–20%). Groups 2 and 3 showed mean 3.4% and 1.4% scar formation, respectively (range 0–12 and 0–5, resp.) (Figure 2). 

## 4. Discussion

This study demonstrated that administration of vitamins E and C during development of acute pyelonephritis can reduce severity of scar formation. Pyelonephritis causes renal scar formation. 

It is believed that early antibiotic treatment is of critical importance in minimizing the chance of renal damage and scarring [16]. However, early treatment is often difficult in practice because of delayed clinical presentation. In this study, gentamicin treatment failed to prevent renal tissue damage. Our findings support the idea that delayed antibiotic treatment after 24 h or more is insufficient to prevent renal scaring [16]. Two studies [17, 18] also reported that the addition of anti-inflammatory or antioxidant agents to antibiotic therapy was effective for decreasing renal scaring due to acute PNP, even when treatment was delayed for 72 h. It has been reported that renal damage after acute PNP is more closely related to the extent of the inflammatory process associated with infection than the actual bacterial growth in the kidney [19–21].

 Polyethylene glycol-modified superoxide dismutase (PEG-SOD) and 2-O-octadecylascorbic acid (CV3611) significantly suppressed scarring when administered orally or parenterally during the early stage of kidney infection with MS-piliated bacteria, suggesting that the superoxide and other active oxygens play an important role in renal scarring following infection [22].

Kanter et al. showed that vitamin C treatment alone or with vitamin A may prevent endotoxin-induced renal damage [23]. Similar to our result, antioxidants can protect against diseases and degenerative process caused by oxidative stress.

 Antioxidants can also improve renal function and histological damage produced by CsA administration; thus, antioxidant nutrients could have a therapeutic role in transplant patients treated with CsA [24]. Ajith and coworkers showed that higher doses of vitamins were effective to protect oxidative renal damage and vitamin C was a better nephroprotective agent than vitamin E [25]. 

Antioxidants also have protective effect against other nephrotoxic agents. Some studies indicated that, due to their antioxidant activity, vitamin E and probucol had potential protective effects against GM nephrotoxicity [26–28]. 

In spite of several studies about effectiveness of antioxiadants in preventing pyelonephritis-induced scar formation, our present study had one point: administration of antioxidant can be protective against renal scar formation even without antibiotic. In clinical practice, antibiotic administration is delayed due to vague clinical presentation and/or waiting for result of urine culture. In this situation, we can start vitamins (antioxidants) especially in febrile patients. Antioxidant administration can prevent more renal damage till starting antibiotic after obtaining result of urine culture. 

## 5. Study Limitation

There are some limitations of the present study. First, we did not measure plasma levels of lipid peroxidation. The presented study focused primarily on renal scar formation; therefore, histopathological evaluation of the tissue was accepted as the gold standard. Second, there was the lack of proper control group without any medication. The aim of the present study was evaluation of effect of antioxidant with or without antibiotic administration. Third problem was finding of the dose equivalent of vitamins C and E in humans? When a drug is effective in animal study, based on several follow additional studies, therapeutic index and equivalent dosage for human is calculated. Vitamins are old drug that have been used for many years. Dosages in various diseases and toxic dosage have been defined. So calculation of effective dosage in human based on dosage used in animal for preventing renal scaring is easier.

## 6. Conclusions

The results of this study suggest that administration of antioxidant with or without antibiotic might be beneficial in preventing pyelonephritis-induced renal tissue damage. Although human PNP can be mimicked in the rat [18], additional studies are required to clarify the role of antioxidants for preventing renal scaring in clinical cases and the dose equivalent of vitamins C and E in humans.

## Figures and Tables

**Table 1:** Comparison of microbiologic, chemistry, and pathologic findings between the three groups of Rats.

	Treated with gentamicin *N* = 10	Treated with vitamins E and C and gentamicin = 15	Treated with vitamins E and C *N* = 13	Normal *N* = 5	*P* value
Weight of rats (grams)	205.5 ± 19.7	206.6 ± 34.2	216 ± 15.5	232 ± 13.5	.34
Colony count per gram kidney weight (10^9^)*	2.3	5.9	3.5	0	.21
Mean percent of scar formation (range)	8.6 (0–20)	3.4 (0–12)	1.4 (0–5)	0	.001
BUN (mg/dl)	22.7 ± 2.5	26.7 ± 6.6	24.6 ± 5	24.6 ± 2.6	.194
Creatinine	1.02 ± 0.39	0.7 ± 0.11	0.67 ± 0.16	0.66 ± 0.2	.004
Mean left Kidney weight (grams)	64.2 ± 8.9	66.4 ± 13.3	68.2 ± 10.1	73.4 ± 9.1	.034

BUN: blood urea nitrogen. Values are expressed as mean ± SD.

*48 hours after microbial inoculation.
